# Morphological heterogeneity description enabled early and parallel non-invasive prediction of T-cell proliferation inhibitory potency and growth rate for facilitating donor selection of human mesenchymal stem cells

**DOI:** 10.1186/s41232-021-00192-5

**Published:** 2022-01-30

**Authors:** Yuta Imai, Kei Kanie, Ryuji Kato

**Affiliations:** 1 Department of Basic Medicinal Sciences, Graduate School of Pharmaceutical Sciences, Nagoya University, Tokai National Higher Education and Research System, Furocho, Chikusa-ku, Nagoya, Aichi 464-8601 Japan; 2 Institute of Nano-Life-Systems, Institutes of Innovation for Future Society, Nagoya University, Tokai National Higher Education and Research System, Furocho, Chikusa-ku, Nagoya, Aichi 464-8601 Japan

**Keywords:** Morphological analysis, Mesenchymal stem cells, T-cell proliferation inhibitory potency, Cell manufacturing, Quality control

## Abstract

**Background:**

Within the extensively developed therapeutic application of mesenchymal stem cells (MSCs), allogenic immunomodulatory therapy is among the promising categories. Although donor selection is a critical early process that can maximize the production yield, determining the promising candidate is challenging owing to the lack of effective biomarkers and variations of cell sources. In this study, we developed the morphology-based non-invasive prediction models for two quality attributes, the T-cell proliferation inhibitory potency and growth rate.

**Methods:**

Eleven lots of mixing bone marrow-derived and adipose-derived MSCs were analyzed. Their morphological profiles and growth rates were quantified by image processing by acquiring 6 h interval time-course phase-contrast microscopic image acquisition. T-cell proliferation inhibitory potency was measured by employing flow cytometry for counting the proliferation rate of peripheral blood mononuclear cells (PBMCs) co-cultured with MSCs. Subsequently, the morphological profile comprising 32 parameters describing the time-course transition of cell population distribution was used for explanatory parameters to construct T-cell proliferation inhibitory potency classification and growth rate prediction models. For constructing prediction models, the effect of machine learning methods, parameter types, and time-course window size of morphological profiles were examined to identify those providing the best performance.

**Results:**

Unsupervised morphology-based visualization enabled the identification of anomaly lots lacking T-cell proliferation inhibitory potencies. The best performing machine learning models exhibited high performances of predictions (accuracy > 0.95 for classifying risky lots, and RMSE < 1.50 for predicting growth rate) using only the first 4 days of morphological profiles. A comparison of morphological parameter types showed that the accumulated time-course information of morphological heterogeneity in cell populations is important for predicting the potencies.

**Conclusions:**

To enable more consistent cell manufacturing of allogenic MSC-based therapeutic products, this study indicated that early non-invasive morphology-based prediction can facilitate the lot selection process for effective cell bank establishment. It was also found that morphological heterogeneity description is important for such potency prediction. Furthermore, performances of the morphology-based prediction models trained with data consisting of origin-different MSCs demonstrated the effectiveness of sharing morphological data between different types of MSCs, thereby complementing the data limitation issue in the morphology-based quality prediction concept.

**Supplementary Information:**

The online version contains supplementary material available at 10.1186/s41232-021-00192-5.

## Background

Human-derived mesenchymal stem cells (MSCs) are among the most clinically studied cell sources for advancing cell therapies [1, 2]. Among various therapeutic efficacies explored in MSCs, their anti-inflammatory, immunosuppressive, and immunomodulatory potencies have been widely explored and have been successfully demonstrated in pre-clinical research [3–8]. Recently, owing to their low immunogenicity, MSCs were applied for allogenic treatments, demonstrating their clinical safety and effectiveness [9, 10]. For example, TEMCELL® HS Inj. (JCR Pharmaceuticals Co. Ltd., Tokyo, Japan) is the MSC-based therapeutic product designed for graft versus host disease approved in Japan, which paved the way for allogenic cell-based products [11, 12]. These offer the advantages of off-the-shelf and selection availabilities for objective donor or cell source from a product manufacturing aspect. Consequently, an increasing number of clinical translation research now focus on allogenic usage of MSCs.

Despite the growing demand for MSC-based therapeutic products, their quality control throughout the manufacturing process is still a challenging issue to be overcome [13, 14]. It is known that the potencies of MSCs tend to decay through their in vitro passage culture and often face the unexpected collapse of quality when expansion processes are proceeded [15, 16]. The allogenic usage of MSCs is expected to minimize such manufacturing risks, since it minimizes the risks of unexpected culture errors and efforts for troubleshooting to cover all donor differences, and the process can be optimized or refined easily with quality-assured cell bank. Moreover, with successful donor selection achieving “high potency” lots, not only manufacturing risk reduction but also better therapeutic efficacy with lower manufacturing cost can be expected.

However, as a trade-off for achieving low-risk manufacturing, the establishment of a “good” donor cell bank (master or working cell bank) is a high-cost process with no certain rule for success. Commonly, after the donor screening with criteria of health and infection risks, the collected cells from the screened donors are further expanded and their multiple quality criteria are thoroughly tested. Since such cell bank establishment process is highly intensive with cost and effort, it requires a careful decision to select a candidate lot to be proceeded. If the candidates are too limited to save the cost, it might lose a chance to achieve a high-potency lot that can make large numbers of stocks. In contrast, if too many candidates are proceeded, it results in high cost with risk to have inferior potency banks, which incurs huge financial loss. However, at present, there is no effective evaluation method to predict the promising candidate lots at the early stage. One of the major reasons making such lot selection difficult is that the present cell evaluations are mostly invasive. Although, ideally, multiple evaluation tests are needed to select a promising lot, cells in such donor selection stage are extremely limited and precious. However, invasive methods consume excessive cells, and in case of multiple tests, an enormous number of cells needed to be consumed. Therefore, if early-stage cells can be evaluated with multiple-criteria tests without losing cells, it would be a new approach to facilitate early and efficient donor selection to eliminate risky candidates with low-success rates from the process and help proceed the candidates with higher chances of success in achieving large cell banks with high-potency cells.

Morphology of cells had been used as an important signature to check cells and is still employed worldwide as a cell status monitoring technique in cell manufacturing. Coupled with advanced image processing, morphological descriptors measured from cell images has been reported to show quantitative correlation with MSC’s quality attributes [17–26]. Moreover, the application of machine learning has aided in the realization of automatic linkages to utilize the image-derived morphological descriptors to predict the cell quality attributes that are obtained by invasive assays using only images, with high potentials as manufacturing supporting technology [24–26]. Robust and high-performance morphology-based cell quality prediction methods for predicting cell growth rate have been reported in studies [22, 25]. Further, even multiple osteogenic/adipogenic/chondrogenic differentiation potencies that can only be measured at the final stage of cell culture have been predicted in continuously passaged MSCs obtained from early morphological information [19, 20, 22]. Moreover, it has been found that even in the cases where quality attributes cannot be simply defined using single bio-marker measurements, overall anomaly can be detected via comparison of differences of multiple morphological parameters throughout the time course [25, 26]. Taken together, such computer-assisted non-invasive morphology-based quality prediction approaches have high potential to solve the bottlenecks in cell manufacturing.

**Fig. 1:**
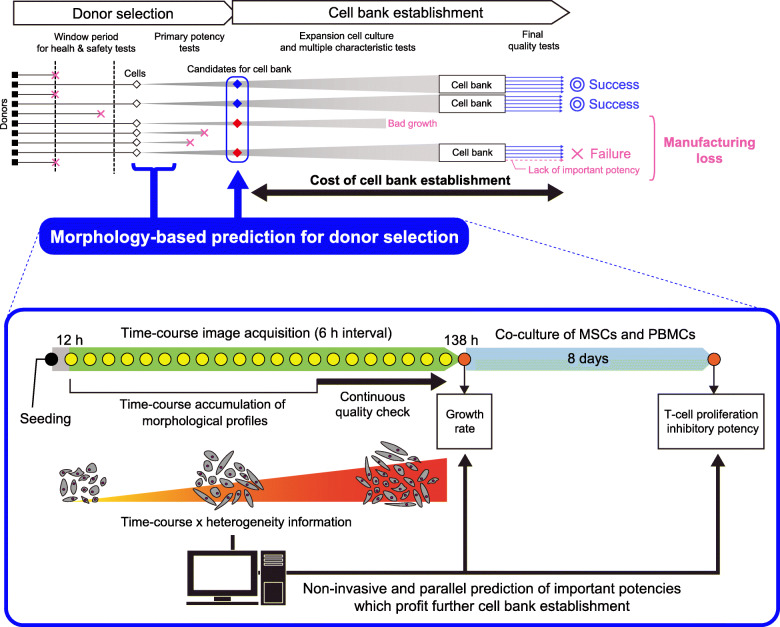
Schematic of morphology-based parallel prediction for growth rate and T-cell proliferation inhibitory potency of MSCs for donor selection. This morphology-based prediction concept is designed to facilitate the donor selection stage to eliminate risky lots for further processing and support the effective cell bank establishment which can pass the final quality tests. The time-course accumulation of morphological profiles which describes morphological heterogeneity enables early and parallel prediction both potencies, and also enables continuous quality check till the final day of culture

In this study, to assist effective donor selection for MSC-based immunomodulatory therapeutic products, we challenged to predict two quality attributes “in parallel” and “in prior” only by using morphological profiles (scheme illustrated in Fig. 1). Escaping from obtaining just a black box image-based prediction models, multiple morphological descriptors were set to describe the cell population heterogeneity transition throughout the time-course to interpret the meaning of used morphological profiles. We have set to predict two quality attributes, the T-cell proliferation inhibitory potency and the growth rate, to show that multiple cell-wasting experimental tests can be replaced by non-invasive direct image-based analysis. If only an image-based prediction can provide both cell potencies at an early stage, it can facilitate minimizing the risk and promote the success rate of lot selection for cell bank establishment. For the model data, we compared 11 lots of MSCs, a mixture of 5 bone marrow-derived MSCs (BMSCs) and 6 adipose-derived stem cells (ADSCs). Morphological profiles were extracted from time-course phase-contrast images, for visualization and prediction of both cell potencies. Subsequently, the modeling methods, types of morphological parameters, and time-window in the construction of prediction models were compared to determine the highest performance and robust model constructing method that enabled early parallel potency prediction. Our study also challenged the use of mixed types of MSCs as training data for such potency prediction, with the aim of finding universally shared morphological characteristics between BMSCs and ADSCs related to quality decay in MSCs, and demonstrate the compatibility of morphological data between different types of MSCs.

## Methods

### Cells and culture

Eleven lots of human MSCs were collected and their detailed information is described in Supplementary Table 1. Six lots of ADSCs from Lonza Japan, Ltd. (Tokyo, Japan) and KURABO (Osaka, Japan), and five lots of BMSCs from Lonza Japan, Ltd., and Lifeline Cell Technology (Frederick, MD, USA) were chosen. The thawed cells from the purchased cryopreserved stocks were directly seeded into two replicates of 6-well plates with 3-well replicates. One plate was used for image acquisition and T-cell proliferation inhibitory assay, and the other for collecting total cells for counting. Such direct seeding without any prior expansion of cells was planned to measure a more direct function of cell lots. For each lot, three independent wells were prepared. All cells were maintained in MSCGM (Lonza Japan, Ltd.) supplemented with BulletKit (Lonza Japan, Ltd.) under conditions of 37 °C and 5 % CO_2_. The purchased PBMCs (Lonza Japan, Ltd., Lot number 0000607334) were stained via 5-(and-6)-carboxyfluorescein diacetate succinimidyl ester (CFSE) using CellTrace™ CFSE Cell Proliferation Kit (Thermo Fisher Scientific, Inc., Waltham, MA, USA) according to the manufacturer’s protocol, counted by Scepter™ (Merk-Millipore, Burlington, MA, USA), and then seeded to the wells of MSCs, which completed the time-course imaging, for co-culture. Total MSCs in the plate for direct counting were harvested by 0.5%-Trypsin/5.3 mM-EDTA Solution (Nacalai tesque, Kyoto, Japan) and their final total cells were counted by Scepter™. Using the cell counts, the PBMC seeding density was set to a ratio of MSC:PBMC = 1:10. Seeded PBMCs were co-cultured with MSCs in RPMI-1640 (FUJIFILM Wako Pure Chemical Corporation, Ltd., Osaka, Japan) for 8 days with 80 μl/well of phytohemagglutinin M-form (Thermo Fisher Scientific, Inc.). According to the result of the T-cell proliferation inhibitory assay, lots 8–11 were designated as “High-risk lots” and lots 1–7 as “Low-risk lots.”

### Measurement of T-cell proliferation inhibitory potency

From the co-cultured well plates of MSCs and PBMCs (assayed in 3 wells), the PBMCs were collected and stained with allophycocyanin (APC)-labeled mouse anti-human CD3 IgG (561810, Becton, Dickinson and Company, Franklin Lakes, NJ, USA) and further fixed via the addition of 1% paraformaldehyde/phosphate-buffered saline according to the manufacturer’s protocol. Thereafter, the fixed cells were placed in an ice bath and subjected to flow cytometry using BD FACS Calibur HG flow cytometer (07B1X00003000012, Becton, Dickinson and Company). Based on a scatter plot of forward scatter vs. side scatter, the gating region was determined, and CD3-positive cells were sorted to obtain their CFSE fluorescent distribution data. Consequently, the ratio of the population with low CFSE fluorescence distribution (proliferated cell population) to the high CFSE fluorescence distribution (unproliferated cell population) was used as the T-cell proliferation score (higher scores indicate lower T-cell proliferation inhibitory potency). Triplicate T-cell proliferation scores were averaged.

### Image acquisition

Regarding the image acquisition, MSCs were seeded to 6-well plates (Corning Incorporated, NY, USA) at 2000 cells/cm^2^ (*N* = 3 wells per each lot) and cultured for 6 days before performing the T-cell proliferation inhibitory potency measurement. Phase-contrast microscopic images of cells were acquired using a BioStation CT (Nikon Corporation) employing a previously reported method of mesenchymal stem cell image acquisition, and tiled images (8 × 8 tiling covering 16 mm^2^, 1000 × 1000 pixels^2^/well) were acquired at 4× magnification. Time intervals were set to 6 h, which began at 12 h following seeding till 138 h. In addition, images containing high noise of debris were eliminated manually.

### Image processing and morphological profiling

All images covering 138 h of culture were processed using the CL-Quant software (Nikon Corporation) employing a processing scheme described in a previous study (Supplementary Fig. 1) [22, 25]. After each single cell region was recognized, 16 basic morphological descriptors (Supplementary Table 2) were measured per cell region in each image, and subsequently, all the measured descriptors were summarized by calculating “mean” and “standard deviation (SD)” using single cells (minimum 200 cells to nearly 10000 cells) captured in an image. Therefore, each image was represented by “mean and SD” of 16 morphological descriptors that summarized all cells in the image. Thus, the complete morphological profile for each condition (= 1 well) comprised 32 parameters (= 16 parameters × (mean and SD)) throughout the time course (22 time points). When examining the effects of morphological profile types, the 32 parameters (designated as “mean + SD”), the majority information represented by mean-related 16 parameters (designated as “without SD”), and the diversity information represented by SD-related 16 parameters (designated as “only SD”) were compared while constructing the prediction models. Further, when examining the time window of parameter usage effect, the time-course data from 12 to 138 h after seeding was shortened from the later period to determine the minimum window size. Moreover, the similarities of multiple morphological parameters representing each sample were visualized employing principal component analysis (PCA) and hierarchical clustering. Thus, using all morphological parameters, samples were colored with their lot label in PCA, whereas in the hierarchical clustering, average linkage clustering with correlation ecoefficiency was used. All analyses and visualizations were performed using R (version 3.4.1) (R Development Core Team, https://www.r-project.org/).

### Construction of prediction models

To construct the T-cell proliferation inhibitory potency classification model, 32 morphological parameters covering the time course of their culture were employed as explanatory parameters, whereas the experimentally determined T cell proliferation inhibitory potency labels (0 for High-risk lots, and 1 for Low-risk lots) were used as objective parameters. To compare the modeling method, least absolute shrinkage and selection operator (LASSO), random forest (RF), and *k*-nearest neighbor (kNN) were compared. To construct the growth rate prediction model, morphological parameters covering the time course were used as explanatory parameters, and cell growth rate was used as objective parameters. In addition, to compare the modeling method, LASSO and RF were compared. The growth rate was calculated considering the image analysis data and defined as the “number of cells counted at 138 h after seeding” divided by the “number of cells counted at 12 h after seeding.” Further, for all model performance validation, leave-lot-out (modification of leave-one-out) cross-validation was applied, and accuracy, precision, and specificity were obtained for classification performances. Furthermore, rooted mean squared errors (RMSE) were obtained to evaluate the performance of quantitative prediction models. Regarding T-cell proliferation inhibitory potency prediction, the acquired 8 × 8 tiling image from each well were divided into four field of views (FOVs) to create 4 pseudo-samples with the same teacher signals, which allowed the increase of the training data size to obtain a more stable prediction model. For cell growth rate prediction, an 8 × 8 tiling image per well was used as one FOV.

### Statistical analysis

To test the difference of cell growth rates and T-cell proliferation scores, data of Low-risk lots were averaged to generate control data, and Dunnett’s test (**p < 0.05) was applied to test the differences of control vs. each High-risk lot. To test the differences between morphological parameters, Student’s *t*-test was applied to test the significance of SD between groups of lots (Low-risk lots vs. High-risk lots).

## Results

### Varieties of cell potencies and their morphologies of 11 MSC lots

To develop morphology-based quality prediction machine learning models, we collected 11 lots of commercially available MSCs for training data, mixing BMSCs and ADSCs comprising various age, gender, and distributors to mimic the diversities of clinically obtained MSCs. Thereafter, we profiled two basic quality attributes that are important for donor selection for manufacturing allogenic MSCs for immunomodulation treatments, their growth rates, and T-cell proliferation inhibitory potencies.

**Fig. 2:**
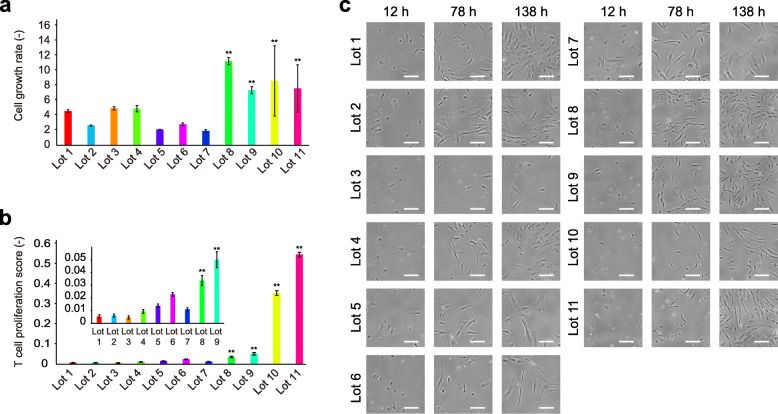
Quality evaluation results of 11 lots of MSCs. **a** Growth rates (*N* = 3). **p < 0.05. **b** T-cell proliferation scores for evaluating T-cell proliferation inhibitory potency (inlet figure is the close of lot 1 to lot 9) (*N* = 3). **p < 0.05. **c** Representative morphological images. White scale bars indicate 200 μm

Considering their growth rates, large potency variations among the lots were found (Fig. 2a), with the greatest difference between lots being more than 5.8-fold, which can greatly affect the manufacturing efficiency and the size of the master cell bank. Further, in our collected MSCs, determining the correlation between the growth rate with the donor information including their origin, age, gender, or manufacturer was challenging.

From the data of T-cell proliferation scores, lots 1–7 indicated extremely low scores, indicating perfect proliferation inhibition. Although such a score indicates these lots passed certain potency threshold, the score could not rank these lots in order. Moreover, since the donor selection stage is an early stage of cell manufacturing, the quality measured at this stage is only supporting information to aid in the decision of further proceeding of cell bank establishment; we decided to focus on discriminating lots with low T cell proliferation inhibitory potencies because it is a risk for further manufacturing. Thus, two lots (lots 10 (ADSC) and 11 (BMSC)) could be considered “clear anomalies” lacking T cell proliferation inhibitory effects (Fig. 2b), whereas considering the CFSE fluorescence peak pattern, lots 8 (ADSC) and 9 (ADSC) were also found to lose some T-cell proliferation inhibitory effects (Supplementary Fig. 2). Thus, it can be categorized as “risky anomalies.” Consequently, we categorized lots 1–7 as “Low-risk lots,” and lots 8–11 as “High-risk lots.” In our collected MSCs, a simple correlation between the T-cell proliferation inhibitory potency with their donor information was challenging to determine.

High-risk lots were found to exhibit high growth rates among the total 11 lots. Therefore, it was assumed that growth rates might be a simple indicator of the lack of T-cell proliferation inhibitory potencies. However, such a rule could not be employed to explain all the lots, such as lots 1, 3, or 4 showing fairly good growth rates. Moreover, conceptually, if we have to simply eliminate good growth rate lots for suspected lack of T cell proliferation inhibitory potency as risks, we must abandon the possibility of obtaining lots with both high potencies. Therefore, simple anti-correlation criteria did not seem to work out and two potency evaluations were considered better to be evaluated independently.

When we compared their morphologies based on the microscopic images (Fig. 2c), distinguishing their quality potencies from the images was challenging. Cell growth in lots 1 (ADSC) and 2 (BMSC) appeared to accelerate in early images; however, their actual growth rate did not exceed that of lots 8–11. Therefore, manual observation of cell morphology or cell growth estimation from a few number of images cannot be considered a reliable evaluation.

### Morphological profiling of MSCs using time-course morphological distribution transition information

To investigate whether such unpredictable MSCs’ potencies can be quantitatively predicted based on only the image-derived morphological information, first, the time-course morphological profiles were quantitated. Thus, 32 parameters of our morphological profile, describing their time-course cell population heterogeneity, were extracted throughout the time-course (22 time points covering 12–138 h after seeding) to objectively compare the morphological differences between lots.

**Fig. 3:**
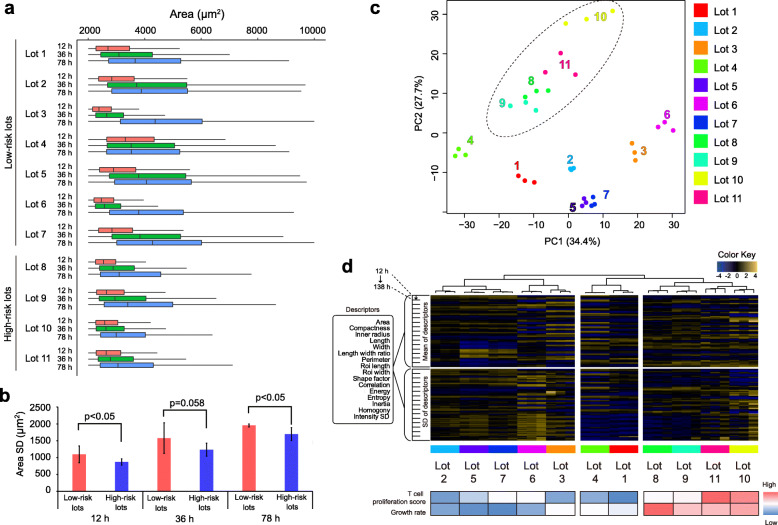
Morphological profile analysis of 11 lots of MSCs. **a** Early time-course transitions of “area” distribution comparing 11 lots. Low-risk lots: Lots 1–7, High-risk lots: Lots 8–11, categorized by the T-cell proliferation inhibitory potencies. **b** Comparison of SDs of “area” among Low-risk lots and High-risk lots at 12, 36, 78 h at their early time-course. Asterisks indicate *p* < 0.05 by Student’s *t*-test. **c** PCA mapping of 11 lots by morphological profiles. Each lot is depicted with the same color dots (3 wells). Dotted circle indicates the cluster of High-risk lots. **d** Hierarchical clustering of 11 lots by morphological profiles. Columns consist of 3 wells × 11 lots, and rows indicate morphological profiles (32 parameters × 22 time points). The heat map under the clustering tree shows their measured potencies (T-cell proliferation score, and growth rate)

In the early time points, the cells remain completely independent of each other; therefore, cellular spontaneous expansion is frequently observed following cell seeding. Therefore, we first compared 11 lots with the early time-course transition of area distribution of 1000 to 4500 cells in images (Fig. 3a), and the High-risk lots were found to exhibit further shrunk distribution when seeded, and less expansion of distribution after 3 days compared to Low-risk lots. Such distribution difference was significant when area SD was compared in each time points (Fig. 3b). Even if it was only focused on a single morphological parameter, such data suggested that there exist morphological differences between Low-risk and High-risk lots. Practically, cells in High-risk lots tend to expand less frequently and rather use their energy for proliferation compared to Low-risk lots. When we focused upon the top 25% of expanded cells in the populations, it was evident that the expanded cells in Low-risk lots expanded to a mean area of approximately 7000 μm^2^ at 78 h, with the High-risk lots sustaining a mean area of approximately 6000 μm^2^ (86 % of Low-risk lots, *p* < 0.05).

To interpret such morphological lot differences from a multi-parametric perspective, the relations between the morphological profiles in 11 lots were visualized by PCA and hierarchical clustering. The PCA visualization (Fig. 3c) first confirmed that the differences between morphological profiles in the replicated wells were much smaller than the lot differences, thus showing clusters formed with the same lots. This indicated that morphological profiles are reproducible data that describe the same conditions. Moreover, such clustering effect was found to be very effective when we compared to the PCA result using only single time-point morphological profiles (Supplementary Fig. 3), indicating that the accumulation of time-course morphological profiles is the key to realize robust morphological comparison of lots. Further, when we overlayed the labels of T-cell proliferation inhibitory potency, the High-risk lots clustered in the PC2-high area with simple unsupervised visualization. The morphological similarities of “cluster of replicates” and “cluster of High-risk lots” were also found in the hierarchical clustering (Fig. 3d), which again indicated that our morphological profiles were robust and reproducible descriptors to determine clusters of High-risk lots. Thus, the result indicated that the cluster containing lots 2–7 can be the cluster to define the representative morphological profile that is shared in lots with T-cell proliferation inhibitory potencies.

### Morphology-based T-cell proliferation inhibitory potency prediction and the effect of morphological parameters

**Fig. 4:**
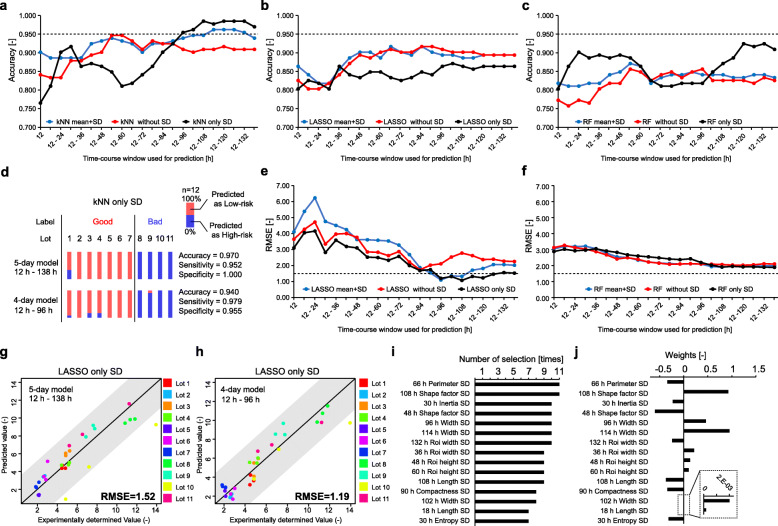
Morphology-based prediction of MSC’s potencies. **a**–**c** Classification performance of T-cell proliferation inhibitory potency compared with modeling methods, type of using morphological parameters, and usage of time-course window size. Samples was classified into two categories: (Low-risk lots) Lots 1–7, (High-risk lots) Lots 8–11. kNN (**a**), LASSO (**b**), and RF (**c**) were compared. Mean + SD: using total 32 morphological parameters combining both mean- and SD-related parameters, Without SD: using mean-related 16 parameters, Only SD: using SD-related 16 parameters. The best model that exceeded the dotted line performance was selected. **d** Comparison of 5-day and 4-day models with each lot prediction results. **e**, **f** Prediction performance of growth rate compared with modeling methods, type of using morphological parameters, and usage of time-course window size. The growth rate (cells in the image at 138 h/cell in the image at 12 h) was used for training each sample. LASSO (**e**) and RF (**f**) were compared. Mean + SD: using a total of 32 morphological parameters combining both mean- and SD-related parameters, Without SD: using mean-related 16 parameters, Only SD: using SD-related 16 parameters. The best model showing RMSE lower than the dotted line performance was selected. **g** 5-day model performance of growth prediction. **h** 4-day model performance of growth prediction. **i** Top 15 parameters selected in the LASSO with only SD model. The number of selections indicates the total number of selections during the leave-lot-out cross-validation. **j** Weights for the top 15 parameters selected in the LASSO with the only SD model

To further extend the efficient morphological profiling effect, we attempted to develop morphology-based potency prediction models based on machine learning. Since it would be profitable to eliminate the risky lots lacking T-cell proliferation inhibitory potency and proceed with the remaining lots as candidates for further expansion, such potency classification models were constructed, and their performances were compared (Fig. 4a–c). In this investigation, three concepts were examined to understand the manner in which the morphological profile can be effectively used in machine learning, and determine the essential information for their performance. Regarding the T-cell proliferation inhibitory potency classification, three types of machine learning methods, linear and non-linear, were compared. To further understand whether the “majority” (mean-related parameters) or “diversity” (SD-related parameters) information contribute more to the prediction, we compared three parameter usages: mean + SD (using all parameters), without SD (mean-related parameters), and only SD (SD-related parameters). Moreover, to investigate the earliest prediction possibility, we changed the time-course window size as the explanatory parameters for the prediction of the same task.

Consequently, it was found that kNN using parameters “only SD” achieved the best performance of prediction after 96 h (Fig. 4a); that is, our best model succeeded in discriminating risky lots lacking T-cell proliferation inhibitory potency only from the morphologies obtained 4 days after seeding (Fig. 4d) and also shorten the 8 days of co-culture assay with PBMCs.

On average, kNN showed the best compatibility for T-cell proliferation inhibitory potency classification. Moreover, although the kNN model using total parameters showed fairly high performance throughout the time course, we considered obtaining high accuracy exceeding 0.950 as important and that it continues to remain stably high after 96 h with the kNN using “only SD,” because high accuracy results in increased profit during manufacturing. Among all models, particularly in the best performing kNN model, it was found that time-course accumulation was extremely important for morphology-based T cell proliferation inhibitory potency prediction. Although the kNN with only the SD model experienced a drop in performance at one point, it achieved stable performance after 96 h, suggesting that “time-course accumulated profile of cellular diversity reflected in SD-related morphology” is the key feature for the prediction. When we observed the time-course profile of representative SD-related morphological parameters, the clear anomaly lots (Lot 10 and 11) were found to exhibit characteristic time-course profile compared to other lots (Supplementary Fig. 4), suggesting that such time-course SD-related parameter profile is characteristic even with single parameters; therefore, multiple combinations achieved in our prediction model exhibited high performances.

### Morphology-based growth rate prediction and the effect of morphological parameters

To further narrow the candidate lots that are predicted to show T-cell proliferation inhibitory potency, their growth rate at 138 h after seeding was predicted considering the earlier morphological profiles. Similar to the T cell proliferation inhibitory potency classification, the effect of machine learning models, parameter types, and time-course window sizes on their prediction performances were compared (Fig. 4e, f). Consequently, among all the models, LASSO with only SD exhibited the highest prediction performance (RMSE < 0.15) after 96 h and maintained its high accuracy till the end (Fig. 4e, g, h). Although earlier window sizes of time-course morphological profiles caused the LASSO model to perform lower than RF, LASSO with only SD was chosen as the best prediction model because the growth rate prediction accuracy greatly affects the cost of manufacturing. Similar to the T-cell proliferation inhibitory potency classification, the “only SD” model again performed the best for future prediction, indicating that the accumulation of SD-related morphology, describing heterogeneity transitions in the cell population, is important information predicting growth as well as T-cell proliferation inhibitory potency. The most frequently selected parameters and its weights in the LASSO with the only SD model indicated that such a high-performance model was combining the information of SD-related morphological information from various time points (Fig. 4i, j), which again implied that “time-course accumulation of morphological diversity information” is important for cell quality prediction, and it should be accumulated for more than 4 days after seeding to render it meaningful.

Combining the results of T-cell proliferation inhibitory potency classification with the growth rate prediction results, our data shows that the high accuracy growth rate prediction model can rank the candidate lots that passed the T-cell proliferation inhibitory potency classification in parallel at day 4 after seeding. Such parallel potency prediction can be applied repeatedly to the sample from 96 to 138 h, overlaying the prediction results to result in higher reliability until the day of cell harvest. In other words, it can be said that as this method is based on label-free image analysis, we can accumulate information to gain high-performance prediction models and can repeat the prediction every day to be confident regarding the predicted results. Such a morphology-based prediction scheme should greatly profit the lot selection process, by enabling multiple evaluations repeatedly without consuming any cells for low-risk cell bank establishment processes.

## Discussion

With the advancement of cell therapy and cell culture technologies, the importance of enabling technologies for non-invasive and continuous in-process cell quality monitoring is expected to accelerate cell manufacturing. Especially, to control the efficiency of the costly cell bank establishment process, image-based donor selection facilitating technology has high potential to eliminate the risky lots at the earliest stage. In this study, we investigated the possibility of non-invasive morphology-based prediction of two basic quality attributes in parallel, T-cell proliferation inhibitory potency and its growth rate, for assisting the donor selection process for allogenic immunomodulatory MSC-based therapeutic products. Our results showed that our proposed morphological profile, which describes cellular heterogeneity and its time-course transition, enables the construction of future potency prediction models that exhibit high performances (accuracy > 0.95 for T-cell proliferation inhibitory potency classification, and RMSE < 1.5 for growth rate prediction) at 4 days after seeding. This achievement shows the potential of non-invasive, early, and data-driven multiple evaluations of precious donor cells for more successful cell bank establishment.

In this work, our prediction was focused on detecting the “risks for further processing”, because risk-based process management is the most recent concept of quality control, known as quality by design (QbD) [27, 28]. Since cells are alive, responsive, and heterogeneous, and, moreover, it is difficult to define their quality by a single measurement, it is now understood that the conventional quality control concept which has led to the chemistry-based manufacturing, known as “quality by testing (QbT),” is not compatible with cells. Simply, even if a quality criterion is measured at some point with partial cells, it never can assure total cell quality nor the final product quality after long processing. Therefore, it is now considered that the total cell culture process should be managed by designing a risk-less process to promise reproducible cell culture. In other words, eliminating risk in each process is the key to successful cell manufacturing. From this aspect, our model eliminates risks of harboring “High-risk lots,” and also low growth rate lots in Low-risk lots; this is an important and novel concept for donor screening as there is no technology currently to support risk-based prior donor selection (scheme illustrated in Fig. 1).

Although our prediction can combine just two quality attribute evaluations simultaneously, it should be noted that such multiple non-invasive evaluations without cell consumption are never possible with the conventional cell assay methods. Moreover, considering that morphology-based cell quality prediction works have shown that it can predict various cell potencies [19–22, 24–26], our prediction technology has the potential to cover more categories of potencies from the same image.

From the viewpoint of “training data preparation”, this study shows that “mixed data of BMSCs and ADSCs” works well for quality prediction. There are reports that indicate that such origin difference renders their potencies different [29–31]. However, with both MSCs, it is also known that the continuous passage and the quality decay rendering the MSCs large and flat is a common phenomenon. Our data, which were obtained via the high-performance potency prediction models using morphology data from both types of MSCs, quantitatively suggest that BMSCs and ADSCs share similar morphological profiles correlating their potency. To the best of our knowledge, such a “morphology-based quality prediction model with the combination data of different origin MSCs” is being reported for the first time, which suggests that we can share and merge morphological data of different tissue origin MSCs in the construction of quality prediction machine learning. Because data accumulation of clinically obtained MSCs in cell manufacturing facilities is limited, morphological data collection for quality prediction model is always a cumbersome task. Our data indicated that we can use the morphological data from other types of MSC culture data. Therefore, for automating such image-based quality control in cell manufacturing facilities, global image data sharing or centralization over different types of MSCs should profit the independent cell manufacturing facilities with small size patients. As there are other types of MSCs reported to possess specialized abilities, such as multi-lineage differentiating stress enduring (Muse) cells [32, 33] and rapidly expanding clone (REC) [34], it is important to investigate the degree to which such different types of MSCs share a universal morphological characteristic that links to their quality. Since machine learning-based image application and its supporting data sharing is evoking [35, 36], it should be profitable for any MSC type to enrich the morphology-based quality prediction models using other MSC information shared worldwide.

Although our data indicated successful machine learning results for MSC’s potency predictions, their risks must be considered as well. The biggest risk in such work is the data quantity used in such machine learning [37]. Shortage of training data quantity can easily result in the machine learning models overfitting. To avoid this, data collection is the bottleneck. However, for every institution, mass producing the MSC data is challenging, which together with punctual time-course imaging was found to be essential in this study, covering numbers of donor cells from the start. Therefore, in the process development stage for manufacturing cell-based therapeutic products, when quality prediction is needed, the machine learning models can only be trained with limited numbers and varieties of lots. However, such models are not fully trustworthy; therefore, it is important to interpret the model structure with unsupervised visualization as shown in our data. If the unsupervised visualization indicates certain efficacies of describing lot differences, and weighted parameters from the machine learning are compatible, the constructed model can be better trusted. This is one of the reasons our prediction modeling did not include deep learning models, which require large training data size and their model structure remains a black box for biologists. Moreover, good varieties of training data for better machine learning must be collected; however, control of such balanced quality variation in the training data is also difficult in MSC data collection, as which donor cell data will be similar or different in prior cannot be identified. Consequently, to tackle such data unbalance issue, we reported one solution of introducing an anomaly detection approach for machine learning to effectively use unbalanced training data in cell morphology machine learning [25, 26]. Our results in the present report added a new feature wherein morphological data can be shared for quality prediction machine learning between different types of MSCs, which implies that such unbalance of data may be covered by morphology data sharing. To expand the morphology-based cell quality prediction methods as a feasible tool for assisting cell manufacturing, this issue of data augmentation will be essential; therefore, our mixed MSC modeling results should lead to further data integration.

In the previous studies, it has been reported time-course morphological profile describing cellular heterogeneity is an effective descriptor for predicting cell growth rate in MSCs [19, 22, 25, 26]. However, the biggest difference between this study and the previous ones is that the present data demonstrated growth rate prediction performances using “cell varieties composed of different lots, including different MSC types,” regardless of whether past prediction models included “cell varieties made of continuous passaged lots.” Therefore, the past prediction models can be fit to predict the growth potency damaged by passage stress. However, in this study, we revealed that our modeling concept and morphological descriptors were effective for predicting growth rates purely from lot difference data.

Consequently, our study indicated that measurement of morphological profiles describing time-course cellular heterogeneity is a key descriptor for predicting both cell growth rate and T-cell proliferation inhibitory potency simultaneously, within 4 days. Such an image-based machine learning model development concept is expected to accelerate the efficiency of process development and minimize the risk to expand the unexpected anomaly cells for product manufacturing. In the future, this technology could be applied to other culture stages and processes since such morphological evaluation is reported to be possible even with spheroids [38].

## Conclusions

In this study, we found that the T-cell proliferation inhibitory potency and growth rate can be predicted early and with high accuracy using only cell morphology information obtained from cell phase-contrast images. However, although our study incorporated certain varieties of MSC lots, further investigation of understanding the gap between the real-world patient-derived variation and the variation in our training data is essential for developing a more robust prediction model. It should also be noted that our study results are limited to the purchasable cellular stage, even if it focuses on the donor screening stage. Although our cells are seeded without expansion from the cryo-stocks to mimic such early cellular status, analysis of more primary MSCs should be performed for improved practicality. Further accumulation of morphological profiles from different types MSCs, combined with the analysis and modeling approach established in this work, should lead to deeper inspection to clarify the universal morphological character shared in MSCs that indicates their potencies.

## Supplementary Information

**Additional file 1: Supplementary Table 1.** Information of MSCs used in this study.

**Additional file 2: Supplementary Figure 1.** Schematic illustration of image processing pipeline.

**Additional file 3: Supplementary Table 2.** Basic morphological parameters measured per single cells.

**Additional file 4: Supplementary Figure 2.** Measured CFSE fluorescence distribution of 11 lots.

**Additional file 5: Supplementary Figure 3.** PCA visualization using single time-point morphological profiles.

**Additional file 6: Supplementary Figure 4.** Time-course transition of a representative morphological parameter area (SD) and shape factor (SD) indicating characteristic time-course profiles between 11 lots.

## Data Availability

The datasets used and/or analyzed during the current study are available from the corresponding author on reasonable request.

## Abbreviations

MSC: Mesenchymal stem cell
BMSC: Bone marrow-derived stem cell
ADSC: Adipose-derived stem cell
PBMC: Peripheral blood mononuclear cell
CFSE: 5-(and-6)-Carboxyfluorescein diacetate succinimidyl ester
APC: Allophycocyanin
PI: Propidium iodide
SD: Standard deviation
PCA: Principal component analysis
LASSO: Least absolute shrinkage and selection operator
RF: Random forest
kNN: *k*-nearest neighbor
FOV: Field of view
RMSE: Rooted mean squared errors
QbD: Quality by design
QbT: Quality by testing
