# Supplementary material for: Morphological heterogeneity description enabled early and parallel non-invasive prediction of T-cell proliferation inhibitory potency and growth rate for facilitating donor selection of human mesenchymal stem cells
Source: Inflamm Regen. 2022 Jan 30;42:8. doi: 10.1186/s41232-021-00192-5 (PMC8801074; doi:10.1186/s41232-021-00192-5)
Supplement: Supplementary file 1 — Additional file 1: Supplementary Table 1. Information of MSCs used in this study. [file 41232_2021_192_MOESM1_ESM.docx]

Supplementary Table 1. Information of MSCs used in this study

AD: adipose tissue, BM: bone marrow

| Lot number | Maker | Lot | Origin | Gender | Age |
| --- | --- | --- | --- | --- | --- |
|  |  |  |  |  |  |
| Lot 1 | KURABO | 00970 | AD | Female | 18 |
| Lot 2 | Lonza | 0000411107 | BM | Female | 21 |
| Lot 3 | Lonza | 0000410257 | AD | Female | 36 |
| Lot 4 | Lonza | 0000439846 | AD | Female | 51 |
| Lot 5 | Lonza | 0000488719 | BM | Female | 41 |
| Lot 6 | Lonza | 0000491129 | BM | Female | 34 |
| Lot 7 | Lifeline cell technology | 03016 | BM | Female | 31 |
| Lot 8 | Lonza | 0000440549 | AD | Female | 44 |
| Lot 9 | Lonza | 0000547705 | AD | Male | 89 |
| Lot 10 | Lonza | 0000421627 | AD | Female | 38 |
| Lot 11 | Lonza | 0000482966 | BM | Female | 23 |
