# Supplementary figures and images for: Morphological heterogeneity description enabled early and parallel non-invasive prediction of T-cell proliferation inhibitory potency and growth rate for facilitating donor selection of human mesenchymal stem cells
Source: Inflamm Regen. 2022 Jan 30;42:8. doi: 10.1186/s41232-021-00192-5 (PMC8801074; doi:10.1186/s41232-021-00192-5)

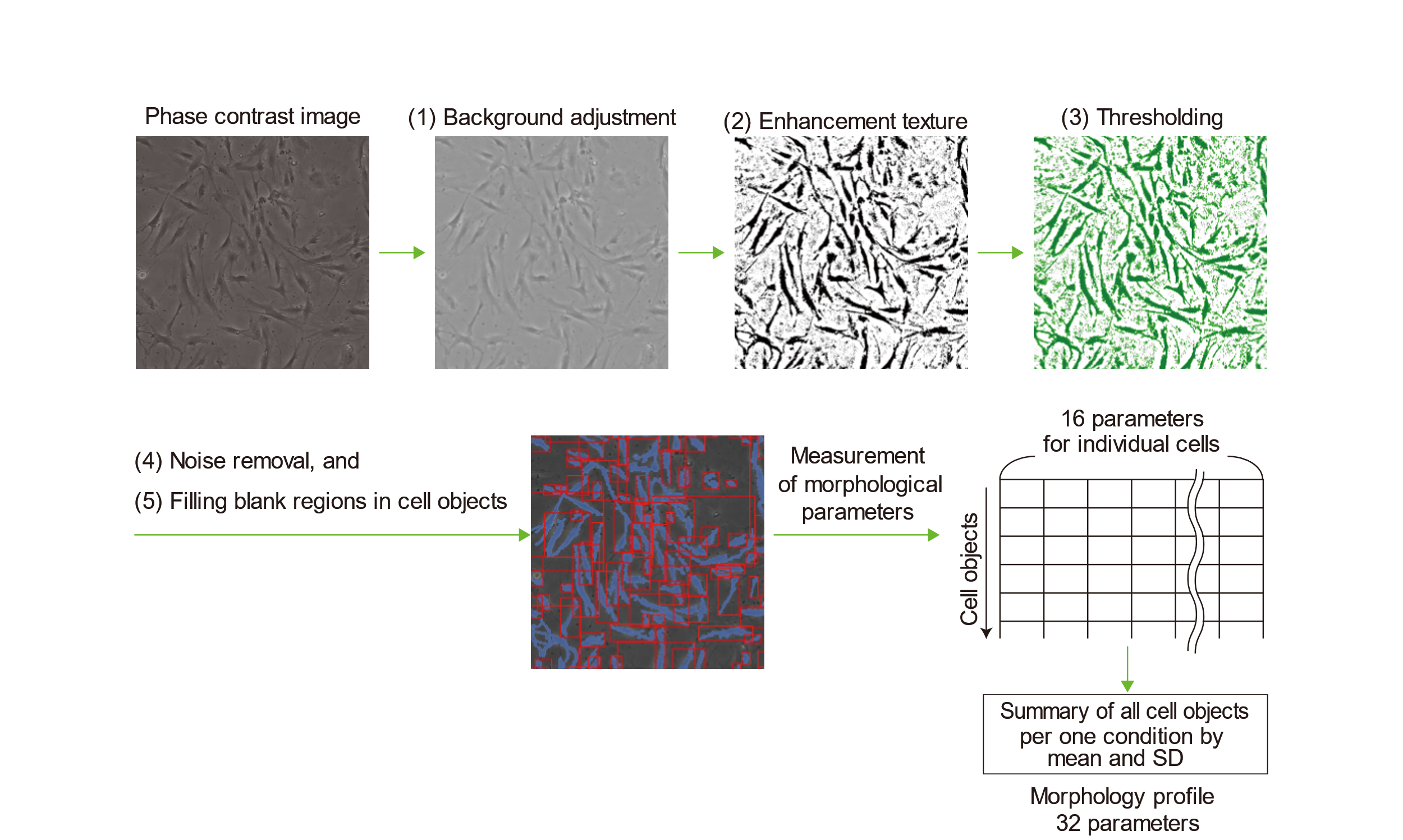


**Supplementary Figure 1:** Schematic illustration of image processing pipeline.

Supplement: Supplementary file 2 — Additional file 2: Supplementary Figure 1. Schematic illustration of image processing pipeline. [file 41232_2021_192_MOESM2_ESM.docx]

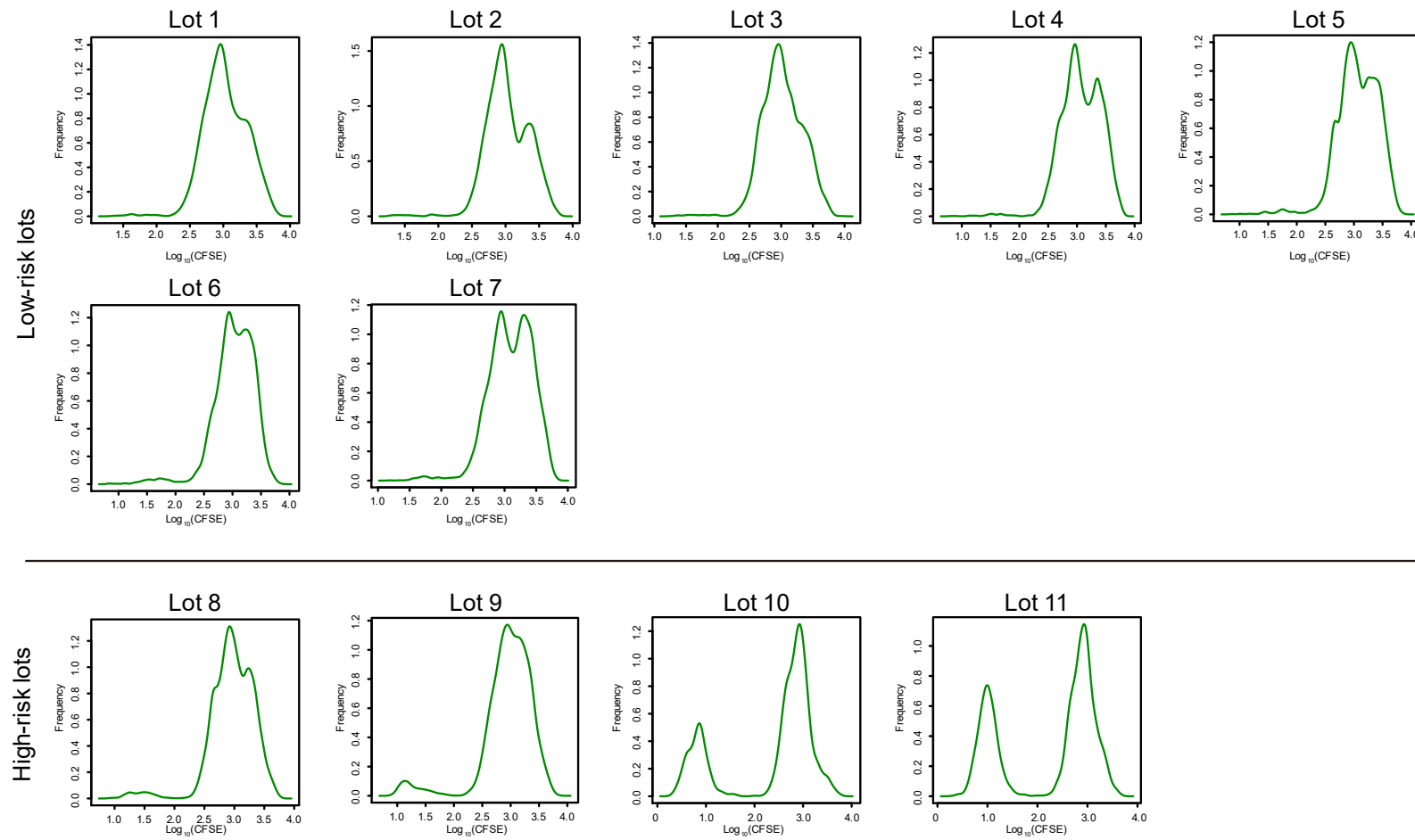

**Supplementary Figure 2:** Measured CFSE fluorescence distribution of 11 lots.

Supplement: Supplementary file 4 — Additional file 4: Supplementary Figure 2. Measured CFSE fluorescence distribution of 11 lots. [file 41232_2021_192_MOESM4_ESM.pdf]

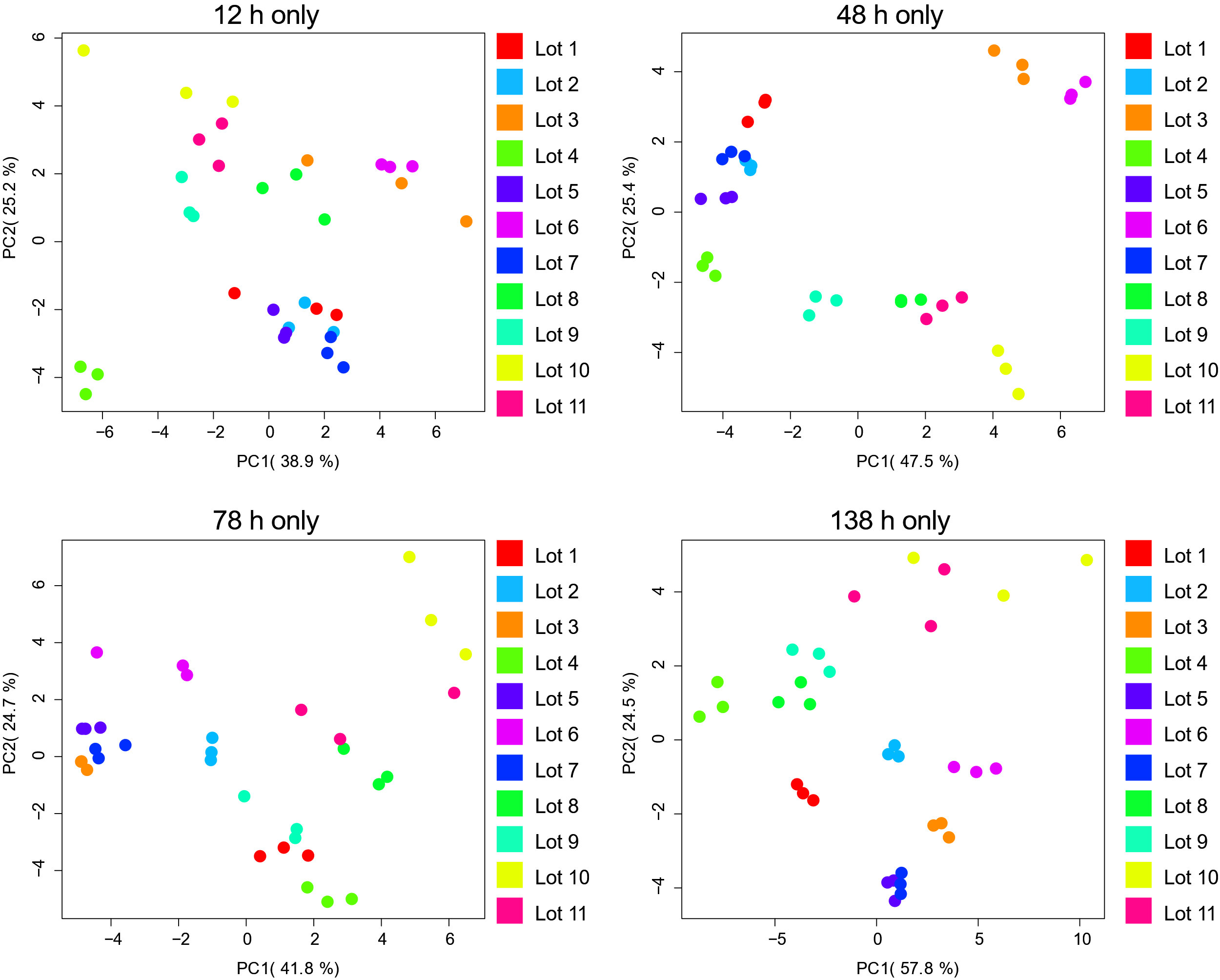


**Supplementary Figure 3:** PCA visualization using single time-point morphological profiles.

Supplement: Supplementary file 5 — Additional file 5: Supplementary Figure 3. PCA visualization using single time-point morphological profiles. [file 41232_2021_192_MOESM5_ESM.docx]
