# Supplementary material for: Morphological heterogeneity description enabled early and parallel non-invasive prediction of T-cell proliferation inhibitory potency and growth rate for facilitating donor selection of human mesenchymal stem cells
Source: Inflamm Regen. 2022 Jan 30;42:8. doi: 10.1186/s41232-021-00192-5 (PMC8801074; doi:10.1186/s41232-021-00192-5)
Supplement: Supplementary file 3 — Additional file 3: Supplementary Table 2. Basic morphological parameters measured per single cells. [file 41232_2021_192_MOESM3_ESM.docx]

Supplementary Table 2. Basic morphological parameters measured per single cells

| Number | Parameter type | Name |  |
| --- | --- | --- | --- |
| 1 | Shape descriptors | Area | Total pixels in the recognized cell region. |
| 2 |  | Compactness | (Perimeter)^2/Area |
| 3 |  | Inner radius | Radius of inscribed circle from the centroid of cell area. |
| 4 |  | Length | Long axis of bounding rectangle covering cell. |
| 5 |  | Width | Short axis of bounding rectangle covering cell. |
| 6 |  | Length width ratio | Length/Width |
| 7 |  | Perimeter | The arc length of recognized cell region. |
| 8 |  | Roi length | Length of Bounding box |
| 9 |  | Roi width | Width of Bounding box |
| 10 |  | Shape factor | 4π(Area)/(Perimeter)^2 |
| 11 | Texture descriptors | Correlation | Gray-Level Co-occurrence Matrix (GLCM) of cell region.  $GLCM=M$  $Correlation=\sum_{i, j} \frac{\left( i-\mu_{x} \right)\left( j-\mu_{y} \right)M_{i,j}}{\sigma_{x}\sigma_{y}}$  $\mu_{x}=\sum_{i, j} jM_{i,j}$  $\mu_{y}=\sum_{i, j} iM_{i,j}$ |
| 12 |  | Energy | Gray-Level Co-occurrence Matrix (GLCM) of cell region.  $GLCM=M$  $Energy=\sum_{i, j} M_{i,j}^{2}$ |
| 13 |  | Entropy | Gray-Level Co-occurrence Matrix (GLCM) of cell region.  $GLCM=M$  $Entropy=-\sum_{i,j} M_{\mathrm{ij}}\log M_{i,j}$ |
| 14 |  | Inertia | Gray-Level Co-occurrence Matrix (GLCM) of cell region.  $GLCM=M$  $Inertia=\sum_{i,j} (i-j)^{2}M_{\mathrm{ij}}$ |
| 15 |  | Homogony | Gray-Level Co-occurrence Matrix (GLCM) of cell region.  $GLCM=M$  $Homogony=\sum_{i, j} \frac{M_{i,j}}{1+\vert i-j\vert}$ |
| 16 |  | Intensity SD | Standard deviation of intensities of pixels in cell region. |
