# Supplementary material for: Morphological heterogeneity description enabled early and parallel non-invasive prediction of T-cell proliferation inhibitory potency and growth rate for facilitating donor selection of human mesenchymal stem cells
Source: Inflamm Regen. 2022 Jan 30;42:8. doi: 10.1186/s41232-021-00192-5 (PMC8801074; doi:10.1186/s41232-021-00192-5)
Supplement: Supplementary file 6 — Additional file 6: Supplementary Figure 4. Time-course transition of a representative morphological parameter area (SD) and shape factor (SD) indicating characteristic time-course profiles between 11 lots. [file 41232_2021_192_MOESM6_ESM.docx]

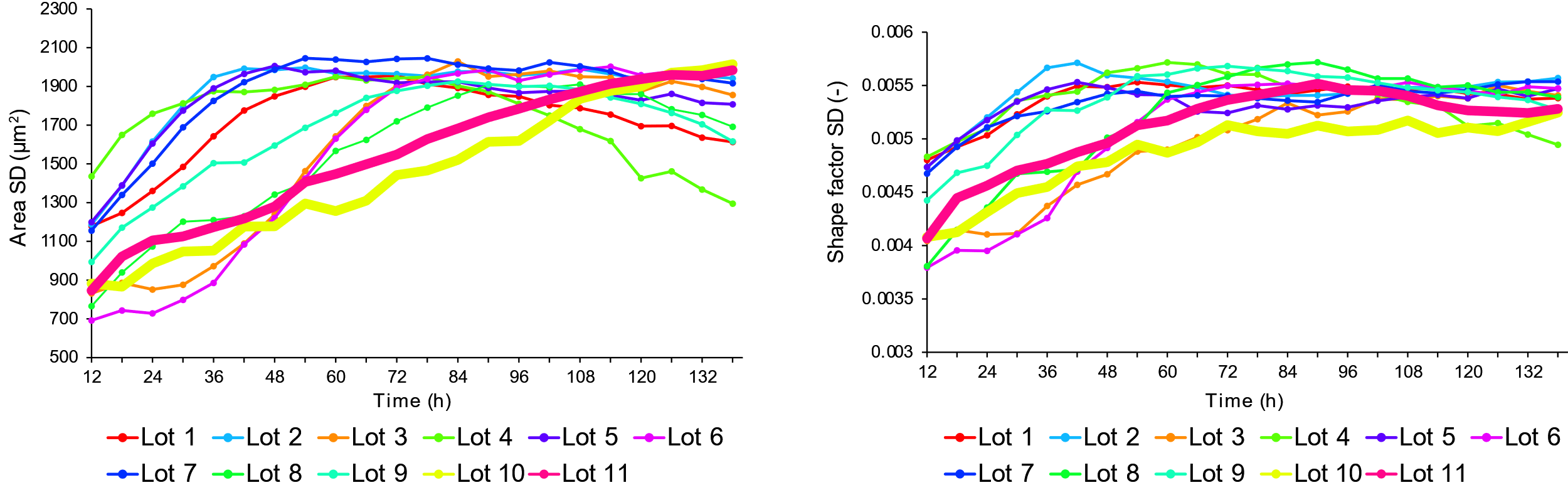


**Supplementary Figure 4:** Time-course transition of a representative morphological parameter area (SD) and shape factor (SD) indicating characteristic time-course profiles between 11 lots.
